# High and Selective Trypanocidal *In Vitro* Response of 1-(2-Methyl-5-nitro-1H-imidazol-yl)-2-phenyl-N-(4-arylthiazol-2-yl)ethanimines Against *Trypanosoma cruzi* INC-5 and NINOA

**DOI:** 10.3390/microorganisms14091947

**Published:** 2026-09-02

**Authors:** Cristoper Ramírez-Sandoval, María-Elena Campos-Aldrete, Benjamín Nogueda-Torres, Rogelio Gómez-Escobedo

**Affiliations:** 1 Laboratorio de Investigación 7 y 10, Departamento de Química Orgánica, Escuela Nacional de Ciencias Biológicas, Instituto Politécnico Nacional, Prolongación de Carpio y Plan de Ayala S/N, Colonia Santo Tomás, Mexico City C.P. 11340, Mexico; christunning1@gmail.com; 2 Laboratorio de Helmintología, Departamento de Parasitología, Escuela Nacional de Ciencias Biológicas, Instituto Politécnico Nacional, Mexico City C.P. 11340, Mexico; bnogueda@yahoo.com (B.N.-T.); rogelio.gomez.14@gmail.com (R.G.-E.)

**Keywords:** *Trypanosoma cruzi*, chagas disease, trypanocidal activity, thiazole, imidazole, molecular docking, trypanothione reductase

## Abstract

Chagas disease remains a major neglected tropical disease with limited therapeutic options and substantial drug-associated toxicity. A previously synthesized series of 1-(2-methyl-5-nitro-1H-imidazol-1-yl)-2-phenyl-N-(4-arylthiazol-2-yl)ethan-1-imines (**5a**–**e**) was evaluated for trypanocidal activity. Molecular docking against *Trypanosoma cruzi* trypanothione reductase (PDB ID: 1BZL) suggested favourable molecular recognition for several derivatives. Compound **5e**, bearing a *p*-nitro-substituted arylthiazole moiety, showed the most favourable predicted binding profile, with a binding energy of −8.41 kcal/mol and an estimated inhibition constant of 0.689 µM. Biological activity was assessed against bloodstream trypomastigotes of two *T. cruzi* isolates representing acute and chronic Chagas disease models, NINOA and INC-5, respectively; however, both isolates were evaluated during acute experimental infection in mice. Compound 5e exhibited the strongest trypanocidal activity, with IC_50_ values of 18.08 and 22.41 µM against NINOA and INC-5, respectively, outperforming nifurtimox and benznidazole under the same experimental conditions. In addition, **5e** exhibited low cytotoxicity in BHK-21 cells (CC_50_ = 2230 µM), yielding selectivity indices of 123 for NINOA and 99.6 for INC-5. Substitution at the *para* position of the arylthiazole ring markedly influenced trypanocidal activity and predicted molecular recognition. Overall, these findings identify the thiazolyl–nitroimidazole scaffold, particularly compound **5e**, as a promising platform for further trypanocidal and mechanistic evaluation.

## 1. Introduction

**Figure 1:**
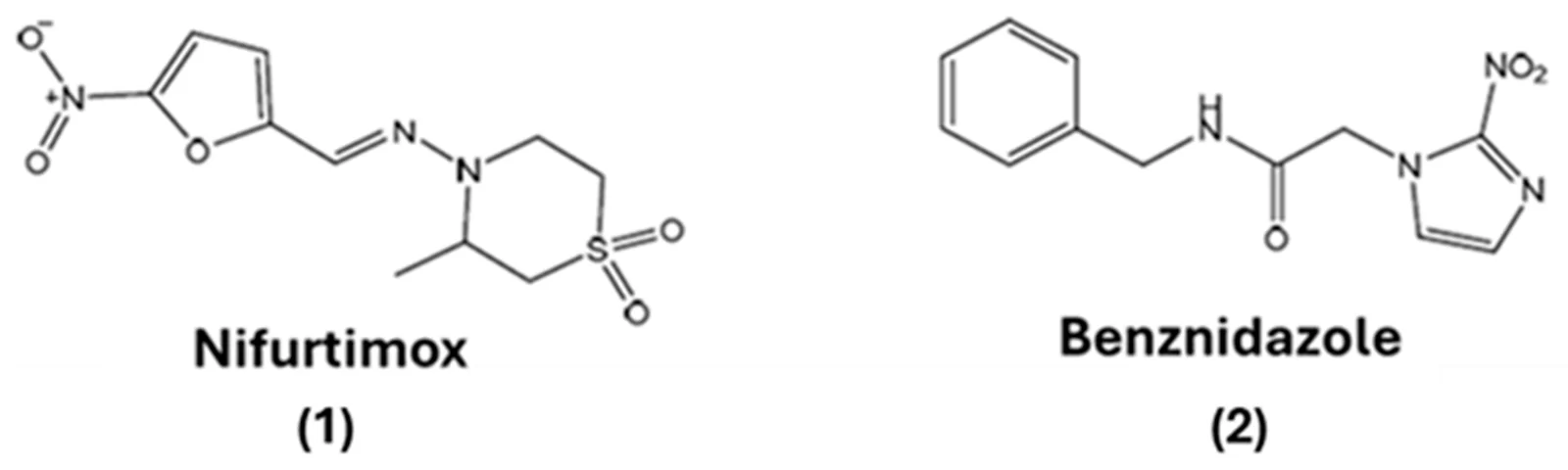
Drugs for the treatment of Chagas Disease.

American trypanosomiasis (Chagas disease) is a zoonotic infection caused by the protozoan parasite *Trypanosoma cruzi*. According to recent estimates, the disease affects approximately 8 million people, places nearly 100 million at risk of infection, and causes around 10,000 deaths annually [1]. Although historically endemic to Latin America, it is now considered a global health concern due to migration and socio-political factors. Transmission occurs via triatomine vectors, congenital infection, oral contamination, blood transfusion, and laboratory accidents [2]. Currently, only two drugs are approved for the treatment of Chagas disease: Nifurtimox (NFT) and Benznidazole (BNZ) (Figure 1) [3].

Although effective in the acute phase, their efficacy decreases with increasing infection duration and patient age [4]. Treatment is also recommended in cases of reactivation (e.g., due to immunosuppression) and for early chronic infection, particularly in women of reproductive age, to prevent congenital transmission [5].

Despite their clinical utility, both drugs require prolonged administration (approximately two months) and are associated with adverse reactions in up to 40% of adult patients [6]. Benznidazole and Nifurtimox are contraindicated during pregnancy and in patients with hepatic or renal impairment. Additionally, Nifurtimox should not be administered to patients with neurological or psychiatric disorders [7,8]. Life-long monitoring for cardiac, digestive, and neurological complications remains necessary [9].

Azole and thiazole cores represent privileged scaffolds in the development of antiparasitic agents [10,11]. Nitroheterocycles, such as the clinically approved benznidazole and fexinidazole, are well-known for their efficacy against trypanosomatids, often acting through nitroreductase-mediated activation pathways. Similarly, isolated thiazole and imidazole derivatives have been widely explored due to their promising bioactivity against *T. cruzi* and other protozoan parasites.

Notably, previous studies by our research group have highlighted the antiparasitic potential of imidazole-fused systems, demonstrating that 2,3-substituted imidazo [1,2-a]pyridines exhibit significant activity against protozoans with a favourable toxicological profile [12].

**Figure 2:**
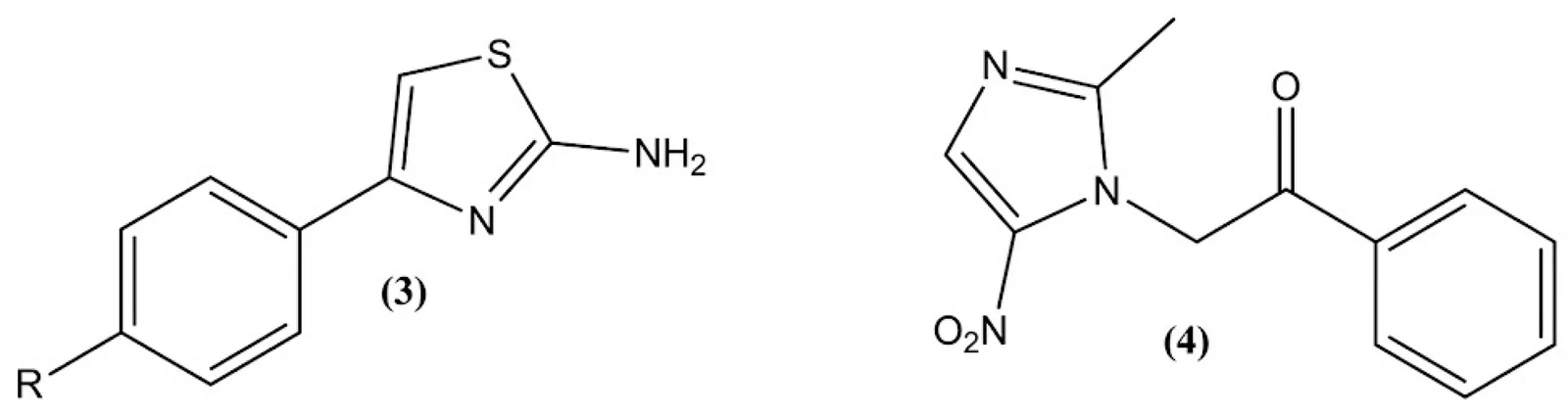
Azaheterocycles of pharmochemical relevance in the construction of antiparasitic agents (**3**) 2-amino-4-arylthiazoles and (**4**) N-phenacyl-2-methyl-5-nitroimidazole.

Furthermore, we recently demonstrated that the structural hybridization of arylthiazole and nitroimidazole moieties yields compounds with highly potent antileishmanial activity, validating the pharmacological potential of this framework against kinetoplastid parasites [13]. In this research, thiazole and imidazole cores were selected as key scaffolds for constructing hybrid heterocyclic systems using 2-amino-4-arylthiazoles (series **3**) and N-phenacyl-2-methyl-5-nitroimidazole (compound **4**) as precursors (Figure 2). The resulting hybrids were designed to assess the contribution of these heterocyclic fragments to the trypanocidal response against *T. cruzi*, while maintaining structural features commonly associated with antiparasitic activity. This strategy also allowed the influence of aryl substitution on biological performance to be evaluated and provided an initial approach to analysing a possible mechanism of action involving enzymatic inhibition.

## 2. Materials and Methods

### 2.1. Biological Models and General Conditions

The biological profiles of the compounds were evaluated in mammalian cell lines and *T. cruzi* bloodstream trypomastigotes. Vero, BHK-21, and CRFK cells were cultured in DMEM supplemented with 6% FBS and 1% penicillin-streptomycin at 37 °C in a 5% CO_2_ atmosphere. For cytotoxicity assays, FBS was reduced to 2%. Trypomastigotes (NINOA and INC-5 isolates, representing acute and chronic models, respectively) were recovered during the acute phase of experimental infection in CD-1 mice and adjusted to 1 × 10^6^ parasites/mL in saline solution. All assays included untreated, vehicle (≤1% *v*/*v* DMSO), and reference drug (nifurtimox and benznidazole; Sigma-Aldrich, Mexico City, Mexico) controls.

### 2.2. Molecular Docking

**Figure 3:**
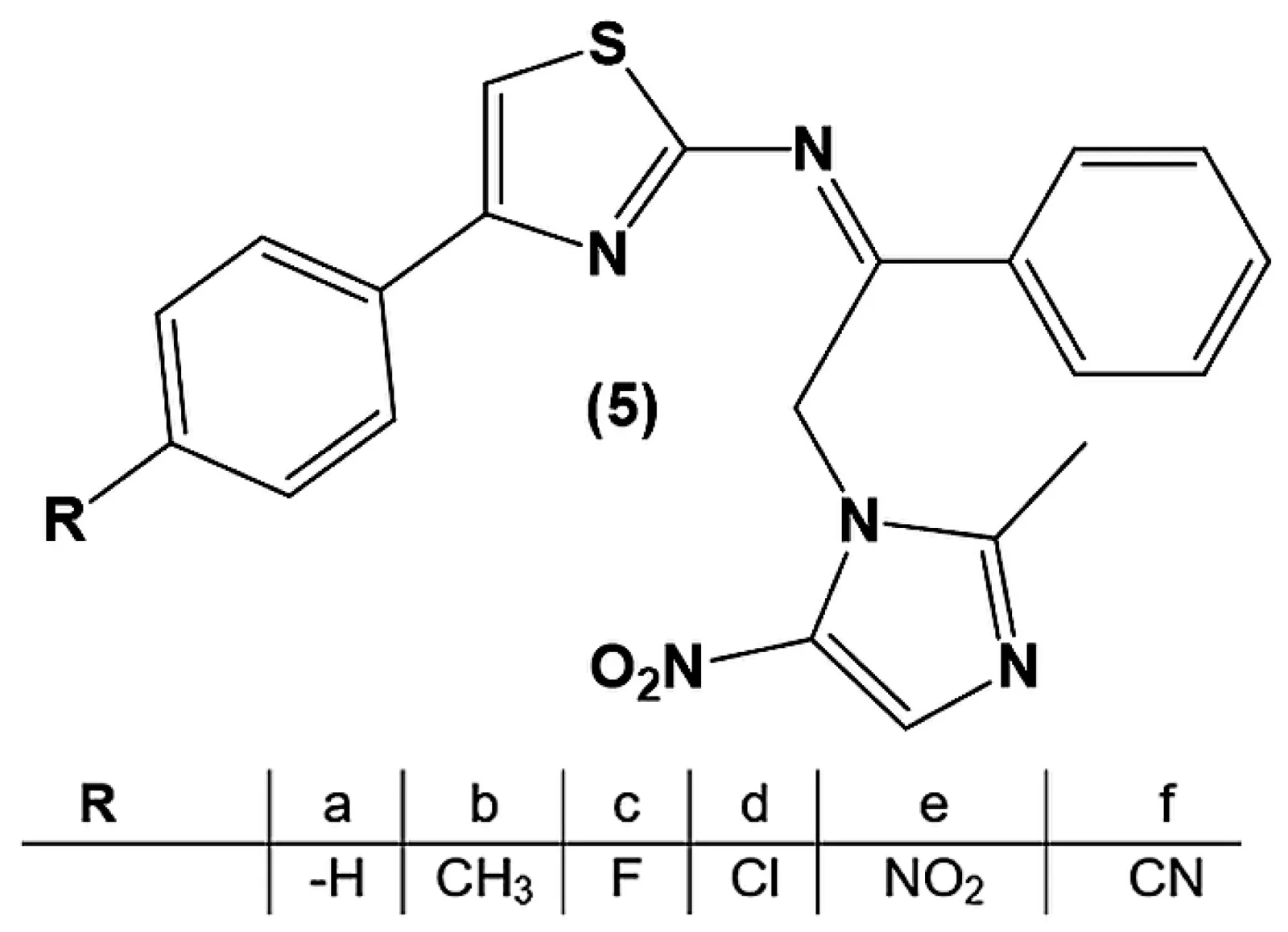
Compounds 1-(2-methyl-5-nitro-1H-imidazol-1-yl)-2-phenyl-N-(4-arylthiazol-2-yl)ethan-1-imines (**5a**–**f** series).

In silico analysis was conducted to estimate the binding affinity and molecular recognition profile of the synthesized azaheterocyclic **5a**–**f** against trypanothione reductase (Figure 3). Compound structures **3a**–**f**, **4**, and **5a**–**f** were drawn in ChemDraw Proffessional 15.0 and geometrically optimized in Avogadro to obtain minimum-energy conformations prior to docking calculations. The crystallographic structure of trypanothione reductase from *Trypanosoma cruzi* (PDB ID: 1BZL) [14] was retrieved from the Protein Data Bank and prepared using AutoDock Tools 1.5.6 by removing water molecules, adding polar hydrogens, and assigning Kollman charges. Site-unspecified docking was performed using a search grid of 160 Å per axis to explore the entire protein surface and identify potential ligand-binding regions without restricting the analysis to the classical catalytic site.

This approach allowed the recognition of catalytic and alternative interaction cavities that may contribute to ligand accommodation. The Lamarckian genetic algorithm was employed with 100 independent runs per ligand to ensure reproducibility and adequate conformational sampling.

The lowest-energy docking pose of each ligand was selected from the AutoDock output and visually inspected using Discovery Studio Visualizer (BIOVIA 2025). The ligand-binding environment and the amino acid residues surrounding each selected pose were identified using the software’s interaction-analysis tools.

### 2.3. Chemical Synthesis

#### 2.3.1. General Procedure for Synthesis of Series 3

The synthesis of 2-amino-4-arylthiazoles (**3a**–**f**) was conducted through a Hantzsch-type cyclization [15]. This process involved the nucleophilic attack of thiourea on the α-halocarbonyl group of *p*-substituted acetophenones (R=H, CH_3_, Cl, F, CN, NO_2_), favouring the formation of a thioimidate intermediate. Subsequent intramolecular heterocyclic closure led to the favoured thiazole core. The compounds of series **3** were isolated by filtration after cooling the medium and purified by ethanolic recrystallization. This protocol consistently provided the desired thiazoles in good to excellent yields (≈85%) with melting points ranging from 148–179 °C, serving as essential building blocks for the next synthetic stage [15].

#### 2.3.2. General Procedure for Synthesis of Series **5**

Compounds of series **5** were obtained following the methodology described by Ramírez-Sandoval et al., 2026 [13], through the condensation reaction between the corresponding 2-amino-4-arylthiazoles (series **3**) and N-phenacyl-2-methyl-5-nitroimidazole (compound **4**). The reaction was carried out under basic conditions in methanol, promoting the formation of the corresponding ethan-1-imine derivatives.

The final products were isolated by direct crystallization and further purified by recrystallization in dichloromethane, affording solids with melting points between 254–271 °C, suitable for structural characterization and biological evaluation. Compound **5f** could not be purified under the established conditions; therefore, it was excluded from the biological studies [13].

The unambiguous structural identity and connectivity of these hybrids were rigorously established via comprehensive IR and 1D/2D NMR spectroscopy (^1^H, ^13^C, COSY, and HMBC), which concurrently confirmed sample homogeneity and the absence of synthetic impurities [13].

### 2.4. Biological Evaluation

#### 2.4.1. Determination of Trypanocidal Effect in *T. cruzi* Trypomastigotes

Bloodstream trypomastigotes were obtained from male CD-1 mice experimentally infected with the NINOA and INC-5 *Trypanosoma cruzi* isolates, associated with acute and chronic Chagas disease models, respectively. In both cases, parasites recovered during the acute phase of experimental infection, when peak parasitemia was reached.

Animals were inoculated with 1 × 10^5^ trypomastigotes/mL and monitored for 2–3 weeks by serial blood-smear examinations until peak parasitemia was detected [16,17]. Blood was collected by cardiac puncture from mice previously anesthetized with sodium pentobarbital, using sodium heparin as anticoagulant [18]. The parasite suspension was subsequently adjusted to 1 × 10^6^ trypomastigotes/mL in physiological saline solution.

An exploratory screening was initially performed at 50 µg/mL. Because this concentration produced extensive parasite lysis and loss of sample integrity, preventing reliable microscopic quantification, concentration–response assays were subsequently performed at five final concentrations ranging from 12.5 to 0.78 µg/mL. For each condition, 90 µL of parasite suspension was dispensed into 96-well microplates and mixed with 10 µL of the corresponding compound solution, resulting in a final assay volume of 100 µL per well. Physiological saline solution was used as the untreated parasite control, whereas DMSO at a final concentration of ≤1% *v*/*v* was included as the compound vehicle control. Nifurtimox and benznidazole were used as reference drugs.

All treatments and controls were evaluated in three independent biological experiments, with each experimental condition tested in technical triplicate. Concentration–response data from each independent experiment were analyzed by Probit regression to estimate IC_50_ values. The results are expressed as the mean ± standard deviation (SD) of the three independent biological experiments.

The plates were incubated at 4 °C for 24 h to preserve the bloodstream trypomastigote stage and minimize parasite differentiation. Parasite quantification was performed using the modified Pizzi–Brenner method [19], and the percentage of lysis was calculated relative to the corresponding vehicle control. Concentration–response data were analyzed by Probit regression to estimate the IC_50_ value of each compound.

#### 2.4.2. Cytotoxicity in Mammalian Cells

The cytotoxic effect of the synthesized compounds was evaluated in three mammalian cell lines: Vero cells (African green monkey kidney epithelial cells), BHK-21 cells (baby hamster kidney fibroblasts), and CRFK cells (Crandell-Rees feline kidney cells). Cells were cultured in DMEM supplemented with 10% fetal bovine serum (FBS) and maintained at 37 °C in a humidified atmosphere containing 5% CO_2_. Prior to the assay, confluent monolayers were washed with phosphate-buffered saline (PBS, 1X), detached with trypsin, centrifuged, and resuspended in fresh medium. Cell counting was performed using a Neubauer hemocytometer according to the trypan blue exclusion method [20].

Cells were seeded in 96-well plates at a density of 3 × 10^5^ cells/well and allowed to adhere for 24 h before treatment. Seven serial dilutions of each compound were prepared from an initial concentration of 800 µg/mL using culture medium as diluent, and each condition was evaluated in triplicate. Cells treated with DMEM containing DMSO at the same final concentration used in the compound dilutions were included as the vehicle control, whereas wells containing only DMEM were used as blank controls.

After treatment, cell viability was determined using a resazurin-based colourimetric assay, which reflects cellular metabolic activity [21]. For plate development, 15 µL of resazurin solution [0.001%] was added to each well, and the plates were further incubated for 24 h at 27 ± 2 °C with 5% CO_2_ atmosphere until stable conversion of resazurin to resorufin was observed. This reduction, mediated by mitochondrial diaphorases in metabolically active cells, generated a signal proportional to the number of viable cells [22].

Absorbance was measured using a microplate reader, corrected against the DMEM blank, normalized to the vehicle control, and expressed as percentage of cell viability. Dose–response curves were analyzed using Probit regression [23], and CC_50_ values were calculated for each mammalian cell line.

Cytotoxicity assays were performed in three independent biological experiments, with each concentration evaluated in technical triplicate. Dose–response curves from each independent experiment were analyzed by Probit regression to estimate CC_50_ values, which are expressed as the mean ± standard deviation (SD) of the three independent biological experiments.

## 3. Results

In rational drug design, evaluating the balance between efficacy and toxicity is essential for identifying promising therapeutic candidates. This pharmacological profile is typically quantified using the half-maximal effective concentration (EC_50_), a broad pharmacological term denoting the concentration of a compound required to induce a 50% response in a biological effect, such as target inhibition, cellular lysis, or toxicity. In the context of this study, this concept is defined in terms of two key metrics: the half-maximal inhibitory or lytic concentration (IC_50_/LC_50_), which measures the *in vitro* trypanocidal efficacy against *T. cruzi*, and the half-maximal cytotoxic concentration (CC_50_), which evaluates toxicity against mammalian host cells. The primary goal is to obtain rationally designed molecules that exhibit high antiparasitic efficacy (low IC_50_) and minimal host–cell toxicity (high CC_50_). To quantitatively evaluate this balance, the Selectivity Index (SI) is established. SI, calculated as the ratio of cytotoxicity to trypanocidal activity (SI = CC_50_/IC_50_), is a critical parameter for assessing the preliminary safety margin and therapeutic viability of the new hybrids.

### 3.1. In Silico Analysis

**Figure 4:**
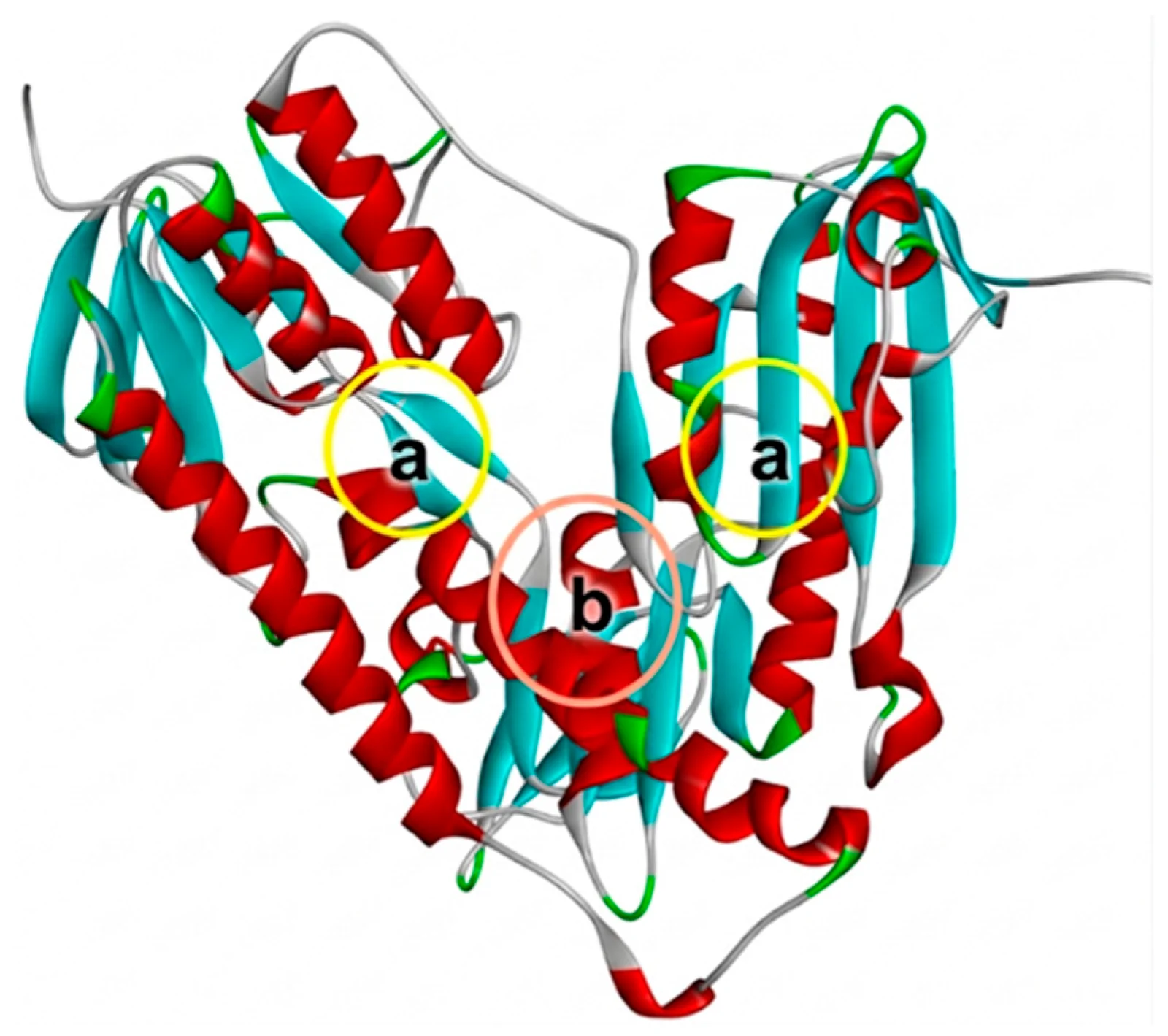
Catalytic sites associated with the protein Trypanothione Reductase (1BZL). In this figure: (a) corresponds to the classical catalytic site and (b) to the non-classical catalytic site.

Molecular recognition analysis of compounds **3a**–**f**, **4**, and **5a**–**f** against trypanothione reductase (1BZL) [14] revealed favorable structural binding, supporting their potential trypanocidal effect. The catalytic sites were analyzed to elucidate their possible mechanism of action. Figure 4 illustrates the 3D organization of trypanothione reductase (PDB: 1BZL) in this dimeric conformation, enabling the identification of its principal catalytic and functional regions.

The catalytic core is defined by the conserved Cys52–Cys57 redox pair, together with structurally and functionally relevant residues such as Tyr198, Ser15, and His461 (a), which participate in electron transfer, substrate positioning, and stabilization of the transition state.

In contrast, the non-classical site (b) corresponds to the dimer interface, a non-catalytic yet functionally essential region characterized predominantly by hydrophobic and aromatic residues, including Phe396, Val361, Ile286, Ala363, and Tyr222. These residues contribute to dimer stability and to the proper spatial arrangement of the active sites. Overall, this structural representation highlights that the enzymatic activity of trypanothione reductase depends on the integration of the redox catalytic core with peripheral regions that modulate the enzyme’s global conformation and dynamics, a characteristic feature of NADPH-dependent redox enzymes in trypanosomatids.

**Figure 5:**
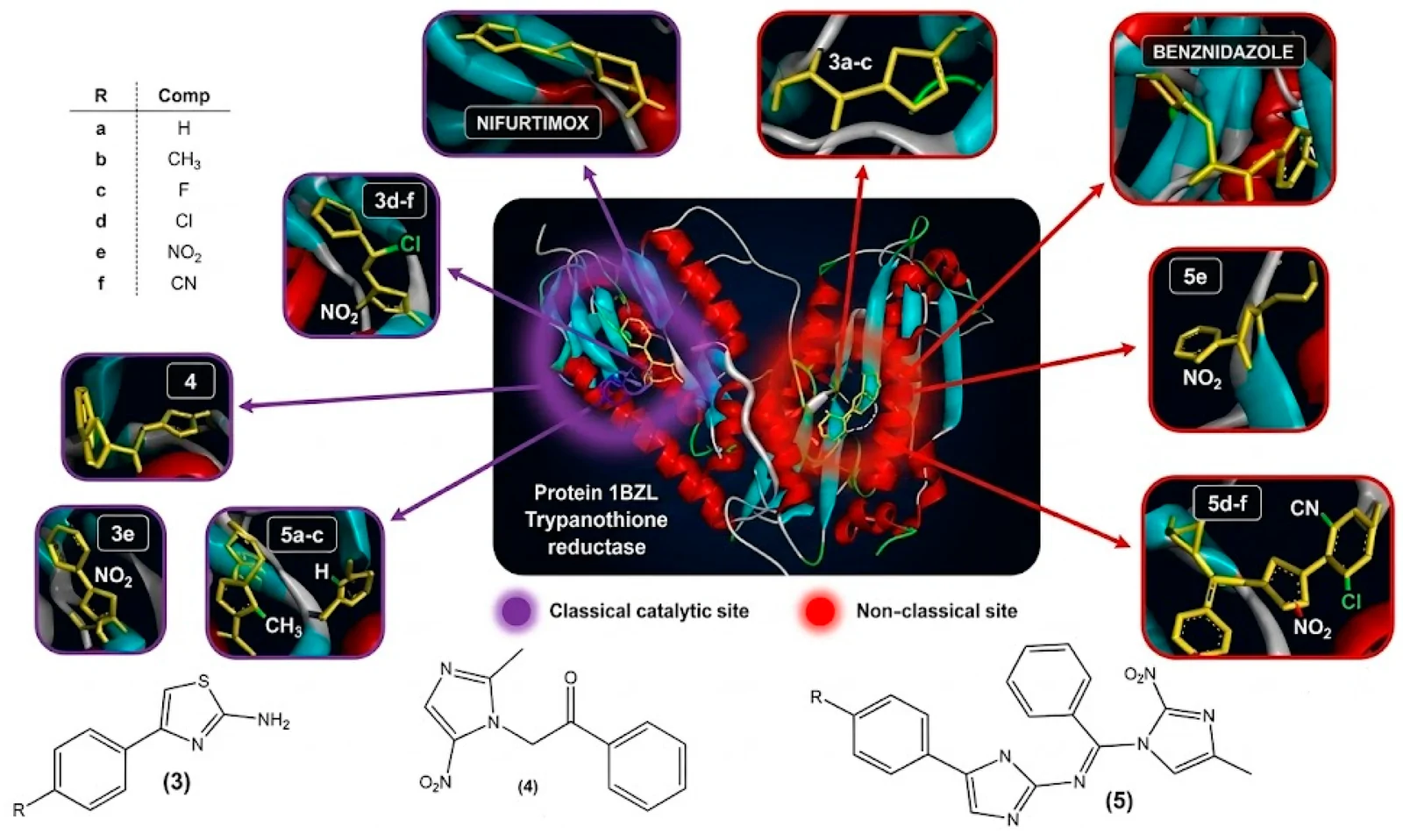
Molecular recognition map showing the interaction patterns of the **3a**–**f**, **4** and **5a**–**f** compounds with trypanothione reductase (PDB ID: 1BZL) [12]. The non-classical binding site is highlighted in red, and the classic site in purple.

Figure 5 presents the molecular recognition map of the evaluated compounds from series **3a**–**f** and **5a**–**f**, in contrast to compound **4**, benznidazole, and nifurtimox, showing their corresponding ligand–protein interaction patterns.

The combined analysis of blind docking and conformational distribution (Figure 5) showed that the arylthiazole hybrids are located at the non-classical binding site of trypanothione reductase (1BZL), outside the main catalytic pocket defined by the Cys52–Cys57–His461–Glu466 amino acid sequence and the typical binding site of the reference drugs. This result is consistent with previous reports describing allosteric cavities in TryR that can accommodate small modulatory molecules and constitute regions distinct from the classical catalytic site [24].

Among these, a pocket adjacent to the NADPH binding site, known as the “doorstop pocket”, and an additional subsite within the trypanothione cavity (Z-site) have been identified as regions susceptible to modulation by non-competitive ligands [25,26].

While Nifurtimox, Benznidazole, and Metronidazole are preferentially accommodated in the central cleft near FAD, the synthesized compounds predominantly occupy lateral hydrophobic grooves and cavities at the dimer interface, regions previously associated with allosteric inhibition and disruption of structural communications between monomers [25,27]. This binding profile is consistent with the affinity values shown in Table 1, where series 5 displayed the most favorable behavior (−∆G = 7.67–8.41; Ki = 6.89 × 10^−3^–2.38 nM), whereas derivatives bearing -H or -Cl showed lower affinities and less stable peripheral accommodation.

In accordance with this binding profile, Table 1 summarizes the recognition mapping obtained for compounds **3a**–**f**, **4**, and **5a**–**f** against trypanothione reductase (1BZL). The affinity constant (−∆G) and estimated inhibition constant (Ki) values reflect differences in ligand recognition among the evaluated series.

The blind docking results indicate that compounds of series **5**, which integrate imidazole and 2-amino-4-arylthiazole (series **3**) pharmacophoric fragments, were not restricted to the canonical catalytic pocket of trypanothione reductase. Instead, these hybrids were mainly accommodated in peripheral and interfacial regions associated with alternative ligand-recognition sites, including the N-terminal domain and the intermonomeric interface. This distribution agrees with previously described noncompetitive or mixed-type inhibition sites in kinetoplastid trypanothione reductase [24,26].

The molecular recognition map further showed that trypanothione reductase exhibits marked conformational plasticity, thereby accommodating ligands in topologically distinct cavities located beyond the classical redox site. The clustering of docking poses near lateral and dimer-interface regions suggests that these cavities may contribute to the stabilization of ligand binding and may influence the relative orientation of protein domains or substrate-access pathways. Therefore, rather than suggesting direct blockade of the catalytic redox center, the docking profile supports a possible allosteric mode of interaction associated with structural modulation of the active dimer.

Consistent with this interpretation, derivatives of series **5** showed the most favourable affinity values, with compound **5e** standing out as the best-ranked ligand (ΔG = −8.41 kcal/mol; Ki = 0.689 nM), followed by **5b**, **5d**, and **5f**, all with Ki values in the low nanomolar range. These findings support the relevance of the imidazole–arylthiazole hybrid scaffold for molecular recognition of trypanothione reductase and provide a structural basis for its observed trypanocidal activity.

To further validate these findings, site-specific docking was performed at the nifurtimox binding pocket, and the results for the synthesized hybrids and reference drugs are summarized in Table 2.

Site-specific docking at the benznidazole binding site confirmed the superior performance of series **5** relative to the reference drugs, series **3a**–**f**, and compound **4**. Compounds in series **5** showed higher binding affinities (ΔG = −7.83 to −8.92 kcal/mol) and inhibition constants in the nanomolar range (Ki = 0.793–2.38 nM), exceeding those of nifurtimox (ΔG = −7.14 kcal/mol; Ki = 5.87 nM) and benznidazole (ΔG = −6.54 kcal/mol; Ki = 33.42 nM).

Among them, compound **5e**, bearing a *p*-NO_2_ substituent, showed the most favourable affinity, followed by **5b** (*p*-CH_3_), **5d** (*p*-Cl), **5a** (-H), **5c** (*p*-F), and **5f** (*p*-CN). This trend indicates that the substituent at the -*p* position modulates ligand recognition within this pocket, likely by influencing the electronic distribution, steric orientation, and hydrophobic accommodation of the arylthiazole fragment.

Post-docking inspection of the lowest-energy pose using BIOVIA Discovery Studio Visualizer placed compound **5e** within a non-classical region of trypanothione reductase, in a local binding environment comprising SerA364, ValA332, PheA231, and ValA328.

These residues are located in peripheral and interfacial regions of the enzyme and define a predominantly hydrophobic environment for ligand accommodation. In contrast, the selected poses of the reference drugs were preferentially located closer to the redox core.

This predicted difference in binding-site preference was consistent with the molecular recognition pattern obtained for **5e**; however, the identified contacts should be interpreted as docking-derived predictions rather than experimentally confirmed ligand–protein interactions.

### 3.2. Source of Compounds

The thiazolyl–imidazole hybrids (series **5**) evaluated in the present study were previously synthesized and structurally characterized by our group as part of an antileishmanial drug-discovery program [13]. In the present work, these compounds were selected to explore their potential trypanocidal activity against bloodstream trypomastigotes of *Trypanosoma cruzi*. By assessing this molecular series in acute and chronic *T. cruzi* infection models and correlating their activity with molecular recognition of trypanothione reductase, this study expands the antiparasitic spectrum of the thiazolyl–imidazole framework beyond its previously reported antileishmanial activity.

The complete spectroscopic suite validating the precise structural framework and the integrity of these derivatives for *in vitro* biological evaluation is available as supplementary data in the previous publication [13].

### 3.3. Trypanocidal Response of the Thiazolyl-Imidazole Hybrids (Series **5**)

#### 3.3.1. *In Vitro* Trypanocidal Response

The trypanocidal activity of compounds **3a**–**e**, **4**, and **5a**–**e** was evaluated against bloodstream trypomastigotes of *Trypanosoma cruzi* obtained from experimentally infected CD-1 mice. The NINOA and INC-5 isolates, associated with acute and chronic Chagas disease models, respectively, were included; however, in both cases, parasites were recovered during the acute phase of experimental infection. This design allowed the trypanocidal performance of the compounds to be compared between isolates of distinct clinical origin under the same experimental conditions. Antiparasitic activity was quantified using the modified Pizzi–Brenner method, and parasite survival was expressed relative to the corresponding untreated controls.

Each concentration was tested in triplicate, and parasite quantification was performed using the modified Pizzi–Brenner method. The percentage of lysis was calculated relative to the corresponding vehicle control. Nifurtimox and benznidazole [28] were included as reference drugs under the same experimental conditions. The LC_50_ values were estimated using Probit regression and served as the primary parameter for comparing the trypanocidal activity of the evaluated compounds against both *T. cruzi* isolates. The concentration–response parameters obtained for compounds **3a**–**e**, **4**, and **5a**–**e** against the NINOA and INC-5 isolates are summarized in Table 3.

During the exploration screening at 50 µg/mL, a generalized lytic effect was observed, characterized by extensive parasite disruption, accumulation of cellular debris, and loss of sample integrity, which prevented reliable microscopic quantification of bloodstream trypomastigotes. Consequently, 50 µg/mL was excluded from the formal concentration–response analysis, and the maximum working concentration was established at 12.5 µg/mL, allowing a more accurate assessment of trypanocidal activity. Qualitative microscopic examination showed a concentration-dependent reduction in parasite survival.

At the highest concentrations within the adjusted range, bloodstream trypomastigotes exhibited progressive loss of motility and morphological alterations, including apparent damage to the cell body and disruption or loss of the flagellum. These changes were more evident for the most active derivatives and were consistent with the lytic response quantified using the modified Pizzi–Brenner method.

Giemsa-stained preparations were examined as a complementary, non-quantitative assessment and suggested alterations in the structural integrity of treated trypomastigotes. Series **5** displayed the highest trypanocidal activity in both models, particularly against INC-5. Within this series, compound **5e**, bearing a *p*-NO_2_ substituent, was the most active derivative, with IC_50_ values of 22.41 µM for INC-5 and 18.08 µM for NINOA, exceeding nifurtimox (187.90 µM) and benznidazole (281.73 µM).

Compounds **5b** (*p*-CH_3_) and **5d** (*p*-Cl) also showed higher activity than the reference drugs, whereas **5c** and **5d** were less active than **5e**. Overall, these observations suggest that the trypanocidal response of the thiazolyl–imidazole hybrids is associated not only with reduced parasite viability, but also with detectable morphological damage in bloodstream trypomastigotes.

#### 3.3.2. Cytotoxic Effect in Mammalian Cells

Prior to the evaluation of trypanocidal activity, the cytotoxicity of the synthesized compounds was analyzed in mammalian cell lines to establish their preliminary safety profile and to provide a reference for selectivity assessment.

This evaluation was especially important for the thiazolyl-imidazole hybrids (series **5**), since nitro-containing pharmacophores are commonly associated with antiparasitic activity but may also contribute to host–cell toxicity.

Accordingly, Vero, BHK-21, and CRFK cells were used as mammalian host–cell models to estimate CC_50_ values and determine whether the compounds exhibited a favorable cytotoxicity profile for calculating their selectivity indices against *T. cruzi*. The results are presented in Table 4.

The cytotoxicity profile of the evaluated compounds in mammalian cells is summarized in Table 3. Overall, all derivatives showed CC_50_ values in the millimolar range, indicating limited cytotoxicity toward non-parasitic cells under the experimental conditions. The hybrid series **5a**–**f** displayed CC_50_ values between 1.89 and 3.05 µM across Vero, BHK-21, and CRFK cells, whereas the arylthiazole precursors from series **3** generally showed higher CC_50_ values, ranging from 3.81 to 7.47 µM. Although hybridization led to a slight decrease in CC_50_ values compared with the individual thiazole precursors, the values remained within a low-cytotoxicity range and were comparable in magnitude to those observed for nifurtimox and benznidazole. Notably, compound **5e**, which was later identified as the most active trypanocidal derivative, exhibited CC_50_ values of 2.01, 2.23, and 2.07 µM in Vero, BHK-21, and CRFK cells, respectively.

BHK-21 cells [29] were selected as the reference mammalian cell line for the subsequent selectivity-index calculation because they represent a well-established, adherent, fibroblast-like, and reproducible host–cell model for standardized *in vitro* assays. This cell line has been widely used for the propagation and experimental evaluation of infectious agents, as well as for mammalian-cell viability studies, supporting its suitability as a reference model under controlled culture conditions [29]. Although no infection assay was performed in the present study, BHK-21 cells provided a consistent mammalian viability reference for estimating CC_50_ values and comparing compound cytotoxicity with trypanocidal activity. Vero and CRFK cells were also evaluated to obtain a broader cytotoxicity profile; however, using BHK-21 cells as the common reference for SI calculation reduces variability associated with cell origin, growth rate, metabolic activity, and sensitivity to xenobiotics.

Therefore, BHK-21 cells were considered only as a reference mammalian viability model, while the full interpretation of selectivity is addressed in the context of the antiparasitic activity data.

#### 3.3.3. Comparative Trypanocidal and Cytotoxic Profile of Compounds **3a**–**e**, **4**, and **5a**–**e**

To integrate the in silico and biological findings, the pharmacological response of compounds **3a**–**e**, **4**, and **5a**–**e** was analyzed by comparing physicochemical, molecular-recognition, trypanocidal, and cytotoxicity-derived parameters. This integrative approach included LogP values, binding affinity toward trypanothione reductase expressed as −ΔG, IC_50_ values obtained against the INC-5 and NINOA models of *Trypanosoma cruzi*, and the corresponding selectivity indices calculated from mammalian-cell cytotoxicity data. The purpose of this analysis was to identify whether the improved trypanocidal activity of the hybrid compounds was associated with favorable molecular recognition and an adequate cytotoxicity margin.

The comparative profile showed that compounds from series **5a**–**e** presented the most favorable behavior, combining higher affinity values, improved trypanocidal activity, and superior selectivity indices relative to the arylthiazoles (series **3**), compound **4**, and the reference drugs, as summarized in Table 5. In particular, compound **5e** emerged as the most promising derivative, exhibiting the best relationships among LogP, −ΔG, IC_50_, and SI values across both infection models.

The comparative profile summarized in Table 5 confirms that the hybridization strategy improved the biological behavior of the evaluated compounds. In general, series **5** showed higher LogP values and stronger predicted binding affinities than the arylthiazole precursors from series **3** and compound **4**, suggesting that incorporation of the nitroimidazole fragment favoured both molecular recognition toward trypanothione reductase and trypanocidal activity. This trend was particularly evident for compounds **5e**, **5b**, **5d**, **5c**, and **5a**, which displayed −ΔG values between 7.67 and 8.41 kcal/mol and lower IC_50_ values than the reference drugs of the evaluated infection models.

Among the hybrid derivatives, compound **5e**, bearing a *p*-NO_2_ substituent, exhibited the most favourable overall profile. This compound combined a balanced LogP value of 4.33, the highest predicted binding affinity of the series (−ΔG = 8.41 kcal/mol), the lowest IC_50_ values against both INC-5 and NINOA models, and the highest selectivity indices. The SI values of 99.60 for INC-5 and 123 for NINOA indicate that the trypanocidal response was observed at concentrations markedly lower than those associated with mammalian-cell cytotoxicity. Compound **5b**, bearing a *p*-CH_3_ substituent, also showed a favourable profile, with IC_50_ values of 36.14 µM and 28.91 µM, and SI values of 65.87 and 82.35 for INC-5 and NINOA, respectively, supporting its relevance as the second most active hybrid.

The remaining series **5** derivatives maintained improved performance compared with the non-hybrid precursors, although with lower activity than **5e** and **5b**. Compounds **5d** and **5c** showed similar trypanocidal activity, with IC_50_ values close to 100 µM, whereas **5a** displayed intermediate activity but a higher SI in the INC-5 model, partly due to its higher CC_50_ value in BHK-21 cells. These results suggest that the nature of the *para* substituent modulates the biological response, with *p*-NO_2_ and *p*-CH_3_ favoring the most favorable balance among molecular recognition, parasite inhibition, and mammalian-cell tolerance.

In contrast, series **3** and compound **4** showed weaker overall profiles. Although these compounds exhibited higher CC_50_ values in BHK-21 cells, their trypanocidal activity was considerably lower, resulting in reduced selectivity indices. The selectivity index was calculated as SI = CC_50_/IC_50_, using BHK-21 cytotoxicity values as the mammalian-cell reference and the IC_50_ values obtained for each *T. cruzi* infection model. Compound **3e**, bearing a *p*-NO_2_ substituent, was the most active derivative among the arylthiazole precursors and showed activity comparable to nifurtimox, but it did not reach the performance observed for the corresponding hybrid **5e**. This comparison supports the thiazolyl–imidazole hybrid framework’s contribution to enhancing the trypanocidal response.

Overall, Table 5 demonstrates that the most promising pharmacological behavior was not determined by cytotoxicity alone, but by the combined relationship among LogP, predicted binding affinity, IC_50_, and SI. The superior profile of compound **5e** supports its selection as the most promising derivative of the series, while compound **5b** represents an additional relevant candidate for further biological evaluation. These findings indicate that the thiazolyl–imidazole scaffold provides a favourable platform for the development of compounds with improved trypanocidal activity and an adequate preliminary selectivity margin.

**Figure 6:**
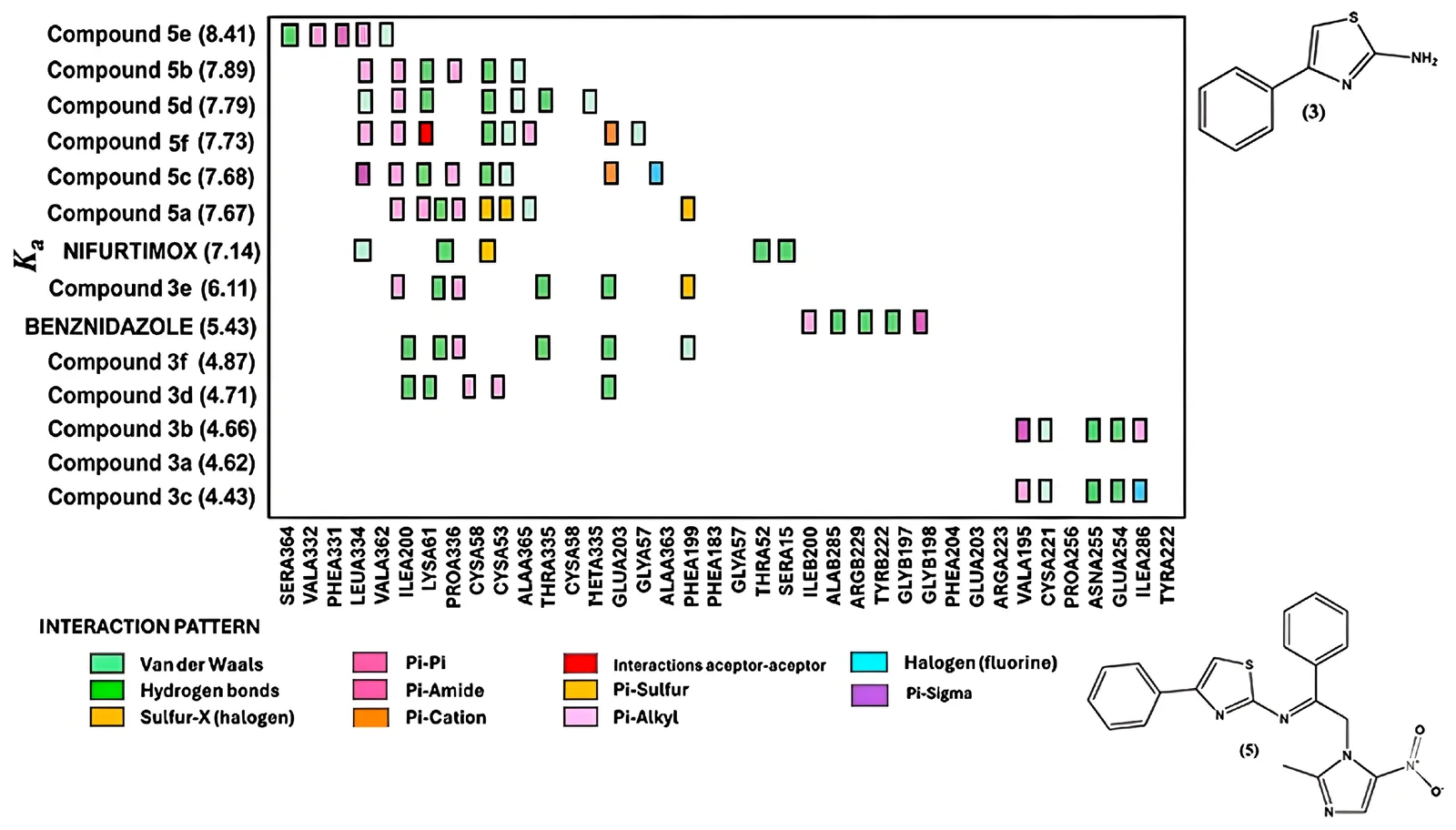
Fingerprint and binding affinities (−∆G) of series **3** & **5** within the active site of trypanothione reductase.

To better compare the molecular behaviour of series **3** and series **5**, an interaction fingerprint analysis was performed within the binding region of trypanothione reductase. This analysis showed that incorporation of the nitroimidazole fragment into the arylthiazole scaffold modified the ligand–protein interaction pattern, favouring stabilizing contacts with key residues of the enzyme. In this context, the interaction patterns shown in Figure 6 provide structural support for the higher affinity energy values observed for the hybrid derivatives of series **5**.

As shown in Figure 6, series **3** and **5** establish a greater number of stabilizing interactions, including hydrogen bonds and π-type contacts, with key active-site residues.

Compared with their arylthiazole precursors (series **3**), the hybrid derivatives (series **5**) showed a broader and more diverse interaction pattern, suggesting that the hybridization strategy contributed to improved molecular recognition and enhanced trypanocidal potential in both infection models.

To further examine these differences, the molecular interaction fingerprints obtained from docking were compared (Figure 6). This analysis showed a well-defined interaction pattern for the synthesized azole derivatives within trypanothione reductase, allowing identification of the structural elements associated with ligand recognition. Among the most active compounds, particularly **5b**, **5d**, and **5e**, recurrent contacts were observed with residues such as ILEA200, LYSA61, and PROA336, mainly through π-type interactions, hydrogen bonds, and van der Waals contacts.

Compound **5e**, which showed the highest binding affinity (−ΔG = 8.41 kcal/mol), displayed the broadest interaction profile, involving residues such as SerA364, ValA332, PheA231, and LeuA334. Figure 6 shows that π–amide and van der Waals contacts were the most recurrent interactions among the active derivatives, whereas halogen, π–cation, π–sulfur, and π–sigma interactions were more selectively distributed. Overall, this interaction pattern suggests that the improved trypanocidal potential of series **5** may be associated with hydrophobic and π-type contacts, together with substituent-dependent interactions that were more evident in the most active derivatives.

**Figure 7:**
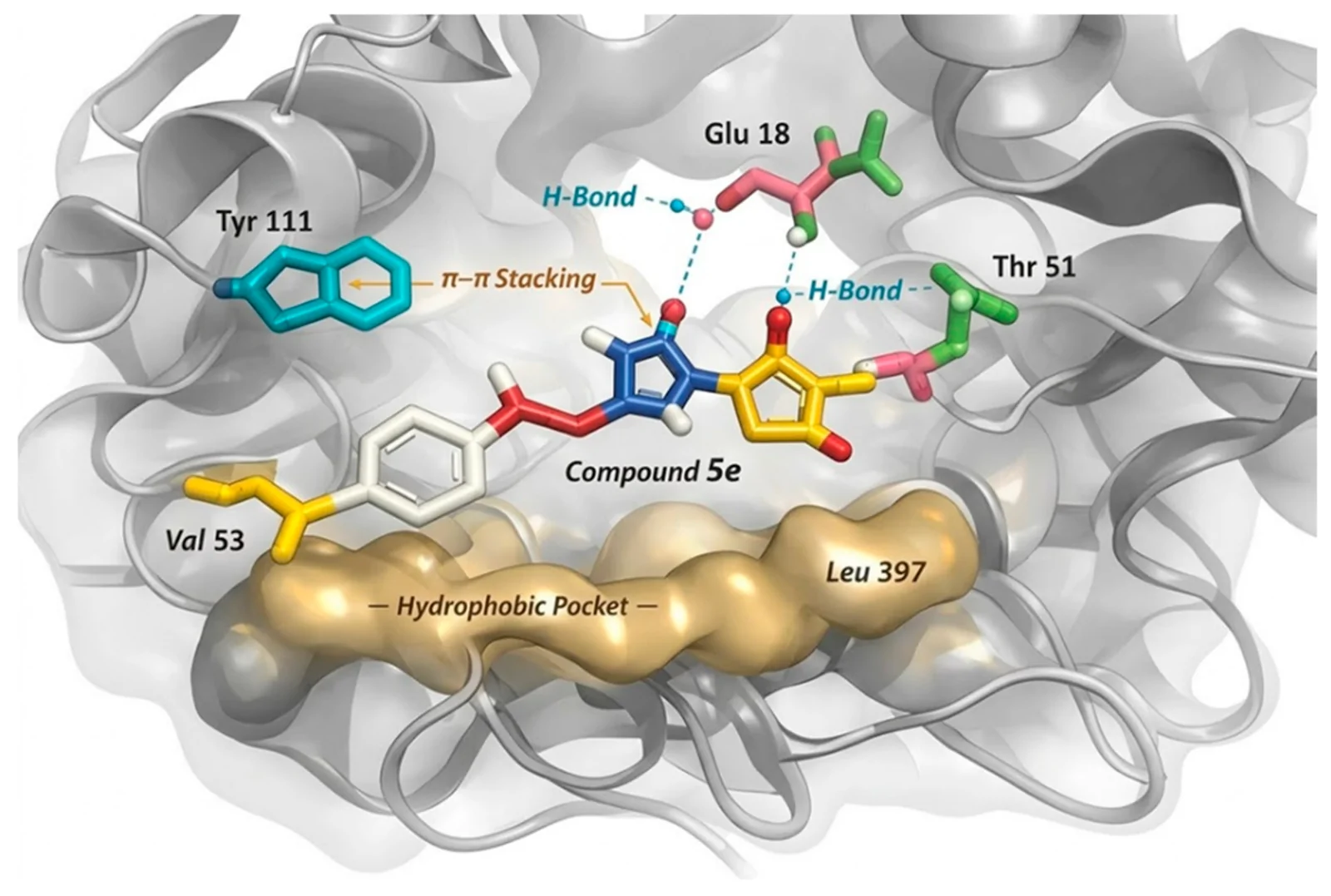
Docking pose of compound **5e** in trypanothione reductase (1BZL), showing π–π stacking with Tyr111, H-bonds with Glu18 and Thr51, and hydrophobic contacts with Val53 and Leu397.

To illustrate the binding mode of the most active compound, the docking pose of **5e** within the catalytic region of trypanothione reductase is shown in Figure 7, highlighting hydrogen-bond interactions with Glu18 and Thr51, π–π stacking with Tyr111, and hydrophobic contacts with residues forming a lipophilic pocket.

To further illustrate the molecular basis of the observed trypanocidal activity, the representative docking pose of compound **5e** within the binding region of trypanothione reductase (1BZL) is shown in Figure 7. The ligand adopts a conformation that allows the establishment of several stabilizing interactions within the enzymatic pocket. Notably, the aromatic system participates in π–π stacking interactions with Tyr111, contributing to the stabilization of the ligand within the catalytic environment.

Additionally, the nitroimidazole and heterocyclic fragments form hydrogen bonds with residues Glu18 and Thr51, reinforcing the anchoring of the molecule within the binding region. The phenyl moiety of the hybrid scaffold extends toward a hydrophobic pocket formed by residues such as Val53 and Leu397, where van der Waals interactions further stabilize the ligand–protein complex.

This combination of polar and hydrophobic interactions supports the strong binding affinity observed in the docking analysis and provides a structural rationale for the favourable trypanocidal profile of compound **5e** compared with the other derivatives of the series, as well as with nifurtimox and benznidazole.

## 4. Discussion

The integrated analysis of binding energies, recognition site topology of trypanothione reductase (1BZL) [14], and biological activity against *T. cruzi* established a robust correlation between the chemical structure of the thiazolyl-imidazole hybrids (series **5**) and their trypanocidal efficacy. With the results presented here, we have shown that the analogous compounds **5a**–**e** exhibited superior affinities compared with series **3** and compound **4**, as well as with the reference drugs nifurtimox and benznidazole. This superior profile was supported by docking analysis, which showed that series **5** exhibited higher affinity (−ΔG = 7.67–8.92) and lower inhibitory constants than the reference drugs nifurtimox and benznidazole, as well as the non-hybrid precursors from series **3** and compound **4**.

This behaviour can be explained by the ability of the hybrid derivatives to interact not only with the main catalytic cleft but also with lateral allosteric cavities and regions adjacent to the active pocket of trypanothione reductase, which have been previously described as sites susceptible to functional modulation [30,31]. In contrast to the reference drugs, which preferentially localized within the catalytic site, series **5** exhibited a bimodal binding pattern that may favour the stabilization of non-productive enzymatic conformations and potentially increase residence time at the molecular target [32,33].

Substitution at the -*p* position of the arylthiazole emerged as a key determinant of pharmacological behaviour. Electron-withdrawing groups such as NO_2_ induce significant electronic redistribution within the conjugated system, optimizing interaction with electropositive regions of the enzyme and enabling more rigid anchoring through dipolar interactions and hydrogen bonding [34]. This effect is directly reflected in the high activity of compound **5e**, the most potent derivative of the series, whose LC_50_ values (22.41 µM for INC-5 and 18.08 µM for NINOA) outperform nifurtimox (187.90 and 161.09 µM) and benznidazole (281.73 and 219.83 µM) by nearly one order of magnitude.

The CH_3_ substituent, although electronically less demanding, increases lipophilicity and reinforces hydrophobic interactions within apolar cavities, explaining the strong performance observed for compound **5b** (LC_50_ = 36.14 and 28.91 µM for INC-5 and NINOA, respectively) [35]. Similarly, fluorine substitution promotes halogen–dipole interactions and inductive effects that stabilize specific orientations at the dimer interface, resulting in intermediate activity.

In contrast, substituents with lower modulatory capacity, such as -H or -Cl, are associated with reduced affinities and higher LC_50_ values, consistent with diminished electronic perturbation of the arylthiazole core.

Additionally, the imine group incorporated into the hybrid scaffold should be highlighted, as it acts as a privileged recognition element in oxidoreductase enzymes and contributes to molecular anchoring through direct electrostatic interactions [36]. Interactions of the conjugated aromatic system further promote accommodation within aromatic clusters of the 1BZL binding site, reinforcing ligand–protein complex stability. Notably, the nitro group exhibits dual behaviour: it exerts a negative inductive effect, facilitating charge-transfer interactions, and may simultaneously mimic functional domains associated with parasite redox pathways, so it is recognized as an important parasitophore and, in this case, also determines trypanocidal activity [37].

The correlation between lipophilicity, enzymatic affinity, and biological activity becomes more relevant when considering pharmacodynamic behaviour across infection phases. Whereas reference drugs display lower LC_50_ values in the NINOA strain (acute phase) and require higher concentrations in INC-5, maybe due to adaptive parasite processes, compounds **5e** and **5b** maintain comparable potency in both models.

Importantly, compound **5e** exhibited outstanding selectivity indices (SI = 99.60 for INC-5 and 123 for NINOA), significantly exceeding those of nifurtimox (SI = 20.42 and 23.82) and benznidazole (SI = 15.29 and 19.59), indicating a favourable *in vitro* selectivity margin. This suggests that the combination of appropriate LogP values (≈4.3–4.8) and a marked tendency to occupy allosteric cavities may partially overcome metabolic barriers characteristic of the chronic phase [38].

Although series **3** exhibits antiparasitic activity due to para-substituted arylthiazole moieties (NO_2_, CN, Cl, F, CH_3_), its overall performance is inferior owing to the absence of the imidazole nucleus, which provides additional molecular recognition and significantly enhances electronic conjugation. This contrast demonstrates that combining the two heterocyclic systems in hybrid series **5** generates electronic and steric synergy that cannot be achieved with either fragment alone. Collectively, these results demonstrate that the trypanocidal activity *in vitro* studies of imidazolyl–arylthiazole hybrids arise from the convergence of multiple structural and physicochemical factors, positioning series **5** as a particularly promising platform for the development of new therapeutic agents targeting *Trypanosoma cruzi*, with a mode of action extending beyond conventional catalytic site inhibition of trypanothione reductase.

## 5. Conclusions

This study shows that the imidazole–arylthiazole hybridization strategy improved the trypanocidal profile of the evaluated compounds relative to their individual precursors and the reference drugs nifurtimox and benznidazole under the same experimental conditions. The combined *in vitro* and in silico results identified series **5** as the most promising group of derivatives. Molecular docking predicted favorable recognition of trypanothione reductase (PDB ID: 1BZL), particularly for the hybrid compounds; however, these findings should be interpreted as computational predictions and require biochemical validation before this enzyme can be established as their molecular target. Docking-pose analysis also suggested possible accommodation in non-classical regions of the enzyme, although an allosteric mechanism of action cannot be confirmed from the present data.

The biological response was influenced by the nature of the para substituent on the arylthiazole ring. Compound **5e**, bearing a *p*-NO_2_ substituent, showed the most favorable overall profile, with LC_50_ values of 22.41 µM against INC-5 and 18.08 µM against NINOA, together with selectivity indices of 99.60 and 123, respectively. Compound **5b** also displayed a favourable trypanocidal and selectivity profile, supporting the relevance of both derivatives for further evaluation.

The activity observed against the INC-5 and NINOA isolates, associated with chronic and acute Chagas disease models, respectively, demonstrates that the thiazolyl–imidazole scaffold maintains trypanocidal activity against isolates of distinct clinical origin. Because bloodstream trypomastigotes from both isolates were recovered during the acute phase of experimental infection, these results should not be interpreted as direct evidence of therapeutic efficacy during chronic infection. Overall, the findings support the thiazolyl–imidazole scaffold as a promising platform for further optimization, mechanistic studies, and expanded biological evaluation against *Trypanosoma cruzi*.

## Figures and Tables

**Table 1:** Binding affinity and inhibition constants obtained from molecular recognition analysis of compounds **3a**–**f**, **4**, and **5a**–**f** toward trypanothione reductase (1BZL).

Compound	* LogP	** (−∆G) (kcal/mol)	** KI (nM)
**5e**	4.33	8.41	0.689
**5b**	4.80	7.89	1.66
**5d**	4.94	7.79	1.96
**5f**	4.11	7.73	2.16
**5c**	5.03	7.68	2.33
**5a**	4.27	7.67	2.38
**NFT (1)**	0.87	7.14	5.87
**3e**	2.35	6.11	33.42
**BNZ (2)**	0.34	5.43	47.2
**3f**	2.15	4.87	105.35
**3d**	3.07	4.71	269.49
**3b**	2.84	4.66	354.2
**3a**	2.79	4.62	401.21
**3c**	2.46	4.62	411.81
**4**	2.54	4.55	570.22

* Calculated in Molinspiration. ** Estimated in Autodock, where −∆G is binding affinity, and KI is inhibition constant.

**Table 2:** Site-specific docking of series **3a**–**f**, **5a**–**f** and compound **4**.

Compound	−∆G (kcal/mol)	KI (nM)
**5e**	8.92	0.793
**5b**	8.03	1.66
**5d**	7.95	1.96
**5a**	7.95	2.16
**5c**	7.85	2.33
**5f**	7.83	2.38
**NFT (1)**	7.14	5.87
**BNZ (2)**	6.54	33.42
**3e**	6.11	47.2
**4**	5.95	105.35
**3f**	4.88	269.49
**3d**	4.71	354.2
**3b**	4.58	385.99
**3a**	4.31	401.21
**3c**	4.2	411.81

**Table 3:** *In vitro* trypanocidal activity of compounds **3a**–**e**, **4**, and **5a**–**e** against bloodstream trypomastigotes of the INC-5 and NINOA *Trypanosoma cruzi* isolates.

Compound	Ar (C-4)	^1^ IC_50_ (µM)	^2^ IC_50_ (µM)
		(INC-5)	(NINOA)
**5e**	NO_2_	22.41	18.08
**5b**	CH_3_	36.14	28.91
**5d**	Cl	108.96	89.70
**5c**	F	104.30	120.80
**5a**	H	89.70	108.96
**3e**	NO_2_	181.21	149.57
**NFT (1)**	-	187.90	161.09
**BNZ (2)**	-	281.73	219.83
**3d**	Cl	825.19	717.83
**3b**	CH_3_	406.63	347.82
**3a**	H	1540.63	1221.29
**3c**	F	1312.52	1070.43
**4**	H	1661.15	1438.96

^1^ IC_50_ determined against bloodstream trypomastigotes of the INC-5 isolate, associated with a chronic Chagas disease model. ^2^ IC_50_ determined against bloodstream trypomastigotes of the NINOA isolate, associated with an acute Chagas disease model. The *in vitro* trypanocidal activity of the evaluated compounds varied with both the molecular scaffold and the nature of the aryl-moiety substituent in the INC-5 and NINOA *Trypanosoma cruzi* isolates, associated with chronic and acute Chagas disease models, respectively.

**Table 4:** *In vitro* cytotoxicity in mammalian cells of the compounds (**3a**–**e**, **4**, **5a**–**e**).

Compound	Ar (C-4)	CC_50_ (µM)		
		^1^ Vero Cells	^2^ BHK-21	^3^ CRFK
**5e**	NO_2_	2010	2230	2070
**5b**	CH_3_	2180	2380	2150
**5d**	Cl	1960	2330	2040
**5c**	F	1890	2390	2070
**5a**	H	2420	3050	2390
**NFT (1)**	-	3360	3830	3170
**3e**	NO_2_	3810	4510	4160
**BNZ (2)**	-	3460	4310	3460
**3d**	Cl	4370	6490	4590
**3b**	CH_3_	4820	5360	4610
**3a**	H	5100	7470	5310
**3c**	F	4790	6970	4960
**4**	H	3220	4890	3680

^1^ Evaluated in Vero cells. ^2^ BHK-21 evaluated in cells. ^3^ evaluated in CRFK cells.

**Table 5:** Integrated pharmacological response of compounds **3a**–**e**, **4**, **5a**–**e**, against *Trypanosoma cruzi*.

Compound	Ar (C-4)	^1^ LogP	^2^ (−∆G)	^3^ IC_50_ (µM)	SI	^4^ IC_50_ (µM)	SI	**^5^ CC_50_**(µM)
				(INC-5)		(NINOA)		
**5e**	NO_2_	4.33	8.41	22.41	99.60	18.08	123	2230
**5b**	CH_3_	4.80	7.89	36.14	65.87	28.91	82.35	2380
**5d**	Cl	4.94	7.79	108.96	21.12	89.70	24.81	2330
**5c**	F	5.03	7.68	104.30	22.91	120.80	19.78	2390
**5a**	H	4.27	7.67	89.70	34.00	108.96	27.99	3050
**3e**	NO_2_	2.35	6.11	181.21	24.87	149.57	30.13	4510
**NFT (1)**	-	0.87	7.14	187.90	20.42	161.09	23.82	3830
**BNZ (2)**	-	0.34	5.43	281.73	15.29	219.83	19.59	4310
**3d**	Cl	3.07	4.71	825.19	7.86	717.83	9.04	6490
**3b**	CH_3_	2.84	4.66	406.63	13.20	347.82	15.43	5360
**3a**	H	2.79	4.62	1540.63	4.85	1221.29	6.11	7470
**3c**	F	2.46	4.62	1312.52	5.31	1070.43	6.51	6970
**4**	H	2.54	4.55	1661.15	2.95	1438.96	3.40	4890

^1^ Calculated in Molinspiration. ^2^ Estimated in Autodock. ^3^ IC_50_ and SI values determined for the INC-5 isolate, associated with a chronic Chagas disease model. ^4^ IC_50_ and SI values determined for the NINOA isolate in an acute Chagas disease model. Both isolates were evaluated using bloodstream trypomastigotes recovered during the acute phase of experimental infection in mice. ^5^ Cytotoxicty in BHK-21 cells.

## Data Availability

The original contributions presented in this study are included in the article. Further inquiries can be directed to the corresponding author.

## Abbreviations

The following abbreviations are used in this manuscript:

BNZ	Benznidazole
BHK-21	Baby Hamster Kidney cells
CC_50_	50% Cytotoxic Concentration
CRFK	Crandell-Rees Feline Kidney cells
H-CRS	Thiazolyl-imidazole hybrids
IC_50_	50% Inhibition Concentration
INC-5	*Trypanosoma cruzi* (chronic infection model).
∆G	Binding affinity
KI	Inhibition constant
LogP	Partition coefficient (Lipophilicity).
NFT	Nifurtimox
NINOA	*Trypanosoma cruzi* (acute infection model).
PDB	Protein Data Bank
PROBIT	Probability Unit (statistical model for dose–response analysis).
SI	Selectivity Index (CC_50_/IC_50_)
TryR	Trypanothione Reductase
